# Eyelid nodule with persistent growth

**DOI:** 10.1016/j.jdcr.2025.07.033

**Published:** 2025-08-30

**Authors:** Magdalena Hoellwerth, Matthias Brandlmaier, Sylvia Selhofer, Martin Laimer, Peter Koelblinger

**Affiliations:** Department of Dermatology and Allergology, Paracelsus Medical University, Salzburg, Austria

**Keywords:** amastigotes, cutaneous leishmaniasis, leishmania infantum, miltefosine, NUCL, sand flies

## History

**Fig 1:**
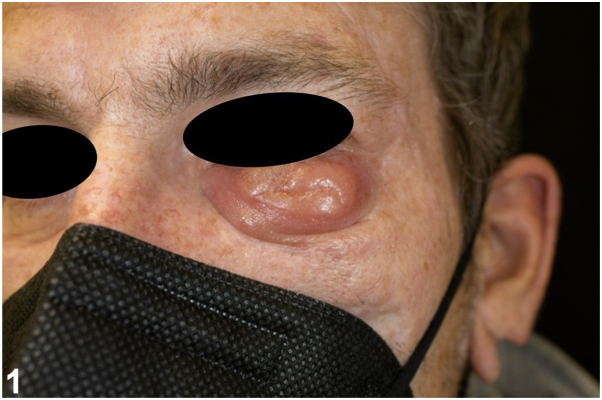
Clinical presentation.

**Fig 2:**
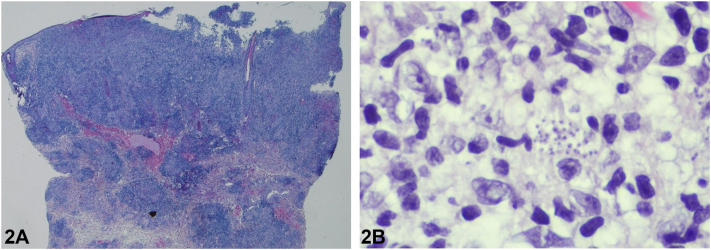
Histology (HE-staining) (**A**) 0.75× magnification and (**B**) 40× magnification.

A 47-year-old, otherwise healthy man was referred for an indolent nodule on his left lower eyelid with a 6-month history of continuous growth. Initially diagnosed as a hordeolum, treatments with antibiotics and corticosteroids (administered locally and systemically) remained ineffective. The lesion was shiny, orange-colored, and lacked ulceration or scaling (Fig 1). No systemic symptoms were noted, and laboratory tests were normal. Travel history was unremarkable. Full skin examination revealed no additional pathologies. Histopathologic examination revealed a skin biopsy showed a dermal infiltrate of predominantly lymphocytes and macrophages with intracytoplasmic basophilic structures (Fig 2). PAS and Grocott stains were negative.

**Question 1: Based on the histological and clinical presentation, what is the most likely underlying diagnosis?**

A. Sarcoidosis
B. Leishmaniasis
C. Necrobiotic xanthogranuloma
D. Histoplasmosis
E. Xanthelasma

**Answers:**

A. Sarcoidosis – Incorrect. Cutaneous sarcoidosis may present as firm, erythematous nodules that occur most frequently in facial areas. The typical histological presentation of sarcoid skin lesions involve non-caseating granulomas composed of epithelioid histiocytes, often accompanied by a rim of lymphocytes in the surrounding. Unlike other granulomas seen in infections or autoimmune diseases, granulomas in sarcoidosis have a relatively low number of lymphocytes, why they are also called “naked granulomas.” The absence of granulomas in histology excludes this diagnosis.
B. Leishmaniasis – Correct. Histopathology consistent with numerous amastigotes in the cytoplasm of macrophages prompted us to perform molecular analyses, which confirmed infection with Leishmania infantum. As chest X-ray and abdominal ultrasound – to exclude systemic involvement – remained inconspicuous, the patient was diagnosed with non-ulcerated cutaneous leishmaniasis (NUCL). Leishmaniasis is a parasitic infection caused by Leishmania species. Species of phlebotomine sandflies are the main vectors of transmission. The clinical appearance of cutaneous lesions is highly heterogeneous, ranging from solitary plaques to multiple, ulcerated, or heavily scaling tumors.^1^
C. Necrobiotic xanthogranuloma – Incorrect. Necrobiotic xanthogranuloma typically show yellowish plaques or nodules. Characteristic histologic features are necrobiotic areas and a granulomatous inflammation as well as the presence of Touton giant cells. The absence of scaling is typical. Necrobiotic xanthogranuloma are often located around the eyes, which fits well with the lesion in the case. However, it can be ruled out histologically due to the intracellular inclusions in macrophages that are present.
D. Histoplasmosis – Incorrect. Histoplasmosis, a fungal infection, is characterized by macrophages with intracellular yeasts in histology. Clinically, cutaneous histoplasmosis may present as papules, plaques, or ulcers, often resembling other granulomatous conditions. Specifically in patients with immunosuppression, cases can be severe, leading to chronic or/and disseminated skin lesions. In this case, however, due to negative Grocott and PAS stains, fungal pathogens could be ruled out.
E. Xanthelasma – Incorrect. Xanthelasma, a benign skin condition, is characterized by yellowish, cholesterol-rich plaques. Eyelids are considered a predilection site for the occurrence of xanthelasma. Typically, the lesions appear in a yellow color, which can be explained by the presence of macrophages filled with lipid, so called “foamy histiocytes.” As there was no infiltration with foamy histiocytes, xanthelasma can be excluded as a correct diagnosis.

**Question 2: Which diagnostic method is most confirmatory for the diagnosis?**

A. Tissue Gram stain
B. Serological antibody tests
C. Polymerase chain reaction (PCR)
D. Direct visualization of flagellated promastigotes in a lesion smear
E. Tzanck smear of the lesion

**Answers:**

A. Tissue Gram stain – Incorrect. In order to detect bacterial infections, Gram staining to identify Gram-positive or Gram-negative organisms is required. Leishmania species are not considered bacteria but protozoan parasites; therefore, amastigotes do not stain with Gram stain.
B. Serological antibody tests – Incorrect. Serological tests, such as ELISA, detect anti-Leishmania antibodies, which are useful in visceral leishmaniasis but have limited sensitivity in cutaneous leishmaniasis. Additionally, prior exposure to Leishmania can lead to false-positive results.
C. Polymerase chain reaction (PCR) – Correct. Leishmania DNA in tissue samples is identified via PCR, a highly sensitive and specific molecular detection method. It allows for species identification, which is crucial for epidemiological tracking and treatment decisions. In this case, PCR confirmed infection with Leishmania infantum, providing evidence of the causative pathogen.^2^
D. Direct visualization of flagellated promastigotes in a lesion smear – Incorrect. Promastigotes are the extracellular and motile form of Leishmania found in the sandfly vector, but not in human tissues. In infected humans, Leishmania exists as intracellular amastigotes inside macrophages, which were observed in this case.
E. Tzanck smear of the lesion – Incorrect. A Tzanck smear is used to diagnose viral infections, such as herpes simplex virus or varicella-zoster virus, by identifying multinucleated giant cells. However, it does not detect Leishmania amastigotes.

**Question 3: What is the most appropriate treatment choice for this non-ulcerated nodule?**

A. Intralesional steroids
B. Miltefosine
C. Surgical excision
D. No treatment, self-limiting
E. Antimalarial drugs (eg, Chloroquine)

**Answers:**

A. Intralesional steroids – Incorrect. Corticosteroids can worsen leishmaniasis by suppressing the immune response, allowing the parasite to proliferate.
B. Miltefosine – Correct. Oral treatment for leishmaniasis includes Miltefosine, an effective drug against *L. infantum*, particularly in cutaneous and visceral infections. It inhibits the phosphatidylcholine synthesis in the Leishmania parasite, disrupting its cell membrane and causing cell death.^3^ Miltefosine was administered in this case and resulted in a complete healing of the skin lesion after 3-months of treatment.
C. Surgical excision – Incorrect. Surgical excision alone is not recommended because it does not address the underlying parasitic infection and may lead to recurrence.
D. No treatment, self-limiting – Incorrect. While some cases resolve spontaneously, persistent lesions require treatment to prevent complications.
E. Antimalarial drugs (eg, Chloroquine) – Incorrect. Antimalarial drugs like chloroquine are primarily used to treat Plasmodium infections that cause malaria by inhibiting the parasite’s ability to metabolize heme. While chloroquine can be effective against malaria and certain autoimmune conditions, it does not have activity against Leishmania parasites.

During the preparation of this work, the author(s) used artificial intelligence including large language models for minor language refinement and improvement of readability in select parts. All contents were critically reviewed and verified by the authors to ensure accuracy. After using this tool/service, the author(s) reviewed and edited the content as needed and take(s) full responsibility for the content of the publication.

## Conflicts of interest

None disclosed.
